# Co-occurrence of approach and avoidance in prolonged grief: a latent class analysis

**DOI:** 10.1080/20008066.2023.2190544

**Published:** 2023-04-04

**Authors:** M. C. Eisma, L. I. M. Lenferink

**Affiliations:** a Department of Clinical Psychology and Experimental Psychopathology, Faculty of Behavioral and Social Sciences, University of Groningen, Groningen, the Netherlands; b Department of Clinical Psychology, Faculty of Social Sciences, Utrecht University, Utrecht, the Netherlands; c Department of Psychology, Health, & Technology, Faculty of Behavioural, Management, and Social Sciences, University of Twente, Enschede, the Netherlands

**Keywords:** Prolonged grief, complicated grief, avoidance, rumination, yearning, continuing bonds, Duelo persistente, duelo complicado, evitación, rumiación, anhelo, lazos continuos, 延长哀伤, 复杂性哀伤, 回避, 反刍, 渴求, 无法终止连接

## Abstract

**Background:** Prolonged grief disorder (PGD) has been included in the International Classification of Diseases (ICD-11) and the Diagnostic and Statistical Manual of Mental Disorders 5 Text Revision (DSM-5-TR). Loss-related avoidance behavior perpetuates grief and effective interventions for prolonged grief symptoms target such avoidance behavior. Yet, behaviors characterized by approach of loss-related cues (i.e. rumination, yearning, proximity seeking) are also implicated in prolonged grief reactions.

**Objective:** To solve this paradox, we will test the Approach Avoidance Processing Hypothesis, which holds that loss-related approach and avoidance behaviors co-occur in PGD, using latent class analyses (LCA).

**Methods:** Two-hundred eighty-eight bereaved adults (92% female) completed questionnaires assessing loss-related approach behaviors (rumination, yearning, proximity seeking), loss-related avoidance behaviors (anxious avoidance, experiential avoidance) and ICD-11 and DSM-5-TR prolonged grief symptoms.

**Results:** LCA demonstrated the best fit for a three-class solution comprising a low approach/low avoidance class (*n* = 98, 34%), a high approach/low avoidance class (*n* = 79, 27%), and a high approach/high avoidance class (*n* = 111, 39%). The latter class showed significantly higher prolonged grief symptom levels and higher odds of probable PGD compared to the other classes.

**Conclusions:** Co-occurrence of loss-related approach and avoidance appears characteristic to prolonged grief reactions. Distinguishing bereaved people with these behavioral patterns from those solely experiencing loss-related approach behaviors may improve the efficacy of PGD therapies.

## HIGHLIGHTS

- A latent class analysis of bereaved adults showed high approach/high avoidance, high approach/low avoidance and low approach/low avoidance classes.
- The high approach/high avoidance class showed highest prolonged grief symptoms and higher odds of probable prolonged grief disorder.
- Co-occurrence of loss-related approach and avoidance appears to characterize prolonged grief.

## Abstract (es)

**Antecedentes:** Los trastornos por duelo persistente (TDP), han sido incluidos en la Clasificación Internacional de Enfermedades (CIE-11), y en el Manual Diagnóstico y Estadístico de los Trastornos Mentales N. 5, Texto Revisado (DSM-5-TR). Las conductas de evitación relacionadas con la pérdida perpetúan el duelo y las intervenciones efectivas para los síntomas de duelo prolongado se enfocan en esas conductas de evitación. Sin embargo, las conductas caracterizadas por un enfoque hacia las señales relacionadas a la pérdida (por ej., rumiación, el anhelo, la búsqueda de proximidad) están también implicadas en las reacciones de duelo prolongado.

**Objetivos:** Para resolver esta paradoja, vamos a probar la Hipótesis de Procesamiento de Aproximación- Evitación, que sostiene que las conductas de aproximación y evitación relacionadas con la pérdida coexisten en TDP, usando un análisis de clase latente (LCA).

**Método:** Doscientos ochenta y ocho adultos en duelo (92% mujeres) completaron cuestionarios que evaluaban conductas de acercamiento relacionados con la pérdida (rumiación, anhelo, búsqueda de proximidad), conductas de evitación relacionadas con la pérdida (evitación ansiosa, evitación experiencial), y síntomas de duelo persistente del CIE-11 y DSM -5-TR.

**Resultados:** LCA demostró el mejor ajuste para una solución de tres clases que comprende una clase de aproximación baja/evitación baja (*n* = 98, 34 %), una clase de aproximación alta/evitación baja (*n* = 79, 27 %) y una clase de aproximación alta/evitación alta (*n* = 111, 39%). La última clase mostró niveles de síntomas de duelo persistente significativamente más altos y mayores probabilidades de TDP en comparación con las otras clases.

**Conclusiones:** La coocurrencia de la aproximación y evitación relacionada a la pérdida parece característico de las reacciones de duelo prolongado. Distinguir a las personas en duelo con estos patrones de comportamiento, de aquellas que solo experimentan comportamientos de aproximación relacionada con la pérdida, puede mejorar la eficacia de las terapias de TDP.

## Abstract (zh)

**背景：**延长哀伤障碍（PGD）已被纳入在国际疾病分类（ICD-11）和精神疾病诊断和统计手册中5文本修订版（DSM-5-TR）。与丧失相关的回避行为使哀伤持续存在，延长哀伤症状的有效干预措施会针对这种回避行为。 然而，丧失相关线索的接近（即反刍，渴求，寻求接近）为特征的行为也与延长哀伤反应有关。

**目的：**为了解决此悖论，我们将使用潜在类别分析（LCA）来检验认为丧失相关接近和回避行为共存的“接近-回避加工假说”。

**方法：**288个丧亲的成年人（92％女性）完成了评估丧失相关接近行为（反反刍，渴求，寻求接近）、丧失相关回避行为（焦虑回避，经验性避免）以及ICD-11和ICD-11和DSM-5-TR延长哀伤症状的问卷。

**结果：**LCA表明了包括低接近/低回避类别（*n* = 98，34％），高接近/低回避类别（*n* = 79，27％）和高接近/高回避类别（*n* = 111，39％）的三类解决方案拟合最好。 与其他类别相比，最后一类表现出显著更高的延长哀伤症状水平和更大的PGD可能患病几率。

**结论：**丧失相关的接近和回避的共存似乎是延长哀伤反应的特征。 区分有这些行为模式者和仅仅经历丧失相关接近行为的人可能可以提高PGD疗法的疗效。

One in ten bereaved persons are estimated to experience severe, persistent and disabling grief, termed prolonged grief, following natural loss (Lundorff et al., 2017) and a higher prevalence is estimated following unnatural loss (49%; Djelantik et al., 2020). The International Classification of Diseases eleventh edition (ICD-11; World Health Organization, 2019) and the text revision of the Diagnostic and Statistical Manual of Mental Disorders 5 (DSM-5-TR; American Psychiatric Association, 2022) have included a diagnosis termed Prolonged Grief Disorder (PGD) characterized by such grief reactions. While these diagnoses share certain similarities with prior proposed grief disorders and each other, they are also qualitatively different (for discussions: e.g. Boelen et al., 2020; Eisma et al., 2020a, 2022; Lenferink et al., 2021). For example, core symptoms of both versions of PGD are severe and persistent yearning and cognitive preoccupation with the deceased, but accessory symptoms show similarities and differences. Moreover, PGD per ICD-11 can be diagnosed at six months post-loss, whereas PGD per DSM-5-TR can be diagnosed at twelve months post-loss. While effective interventions have been developed to treat prolonged grief symptoms, such as cognitive-behavior therapy and complicated grief therapy (e.g. Boelen et al., 2007, 2021; Bryant et al., 2014; Rosner et al., 2014; Shear et al., 2005, 2016), average effect sizes are modest (for a meta-analysis: Johannsen et al., 2019). Understanding malleable mechanisms that lead to the persistence of PGD therefore appears important to improve treatments.

One mechanism assumed central to prolonged grief is loss-related avoidance (Boelen et al., 2007; Shear et al., 2007). Some bereaved people are inclined to avoid objects, situations, and/or memories that remind them of painful aspects of the loss. Such avoidance is proposed to hinder the integration of memories of the loss within the existing autobiographical knowledge base, leading to prolonged grief. Indeed, avoidance of loss-related cues relates positively and strongly to prolonged grief symptoms (for a review: Eisma & Stroebe, 2021). Moreover, interventions focused on countering these avoidant tendencies, i.e. exposure therapy, appear particularly effective in reducing prolonged grief symptoms, both as an added treatment component (Bryant et al., 2014) and as a stand-alone treatment (Boelen et al., 2007; Eisma, Boelen, et al., 2015).

Indeed, the empirical support for a role of loss-related avoidance in PGD is relatively well established. Yet, this support appears at odds with the observation that emotional, cognitive and behavioral processes seemingly signaling loss-related approach also characterize severe grief reactions. First, across diagnostic systems, yearning, defined as an intense, unsatisfied, and future-oriented appetitive desire for the lost person (Davis et al., 2003), is a core symptom of PGD (ICD-11; World Health Organization, 2019; DSM-5-TR, American Psychiatric Association, 2022). Yearning relates to heightened Nucleus Accumbens activation, a reward area of the brain, in people with prolonged grief reactions while they are looking at pictures of the deceased (O’Connor et al., 2008; see Kakarala et al., 2020 for a review of support for a role of the brain’s reward system in PGD). Second, engaging in behavior to retain a connection with the deceased, such as cherishing objects belonging to the deceased, visiting the grave, or looking at old pictures (called proximity seeking or continuing bonds) is more common among people with higher prolonged grief symptoms and predicts the persistence of grief over time (Boelen et al., 2006a; Field et al., 2003). Third, rumination focused on the loss (e.g. repetitive thoughts on why the death happened and how one could have prevented it) occurs more frequently among those with probable PGD (Doering et al., 2018) and worsens prolonged grief symptoms over time (e.g. Eisma, Rinck, et al., 2015; Eisma et al., 2021).

Together, this yields what we will term *grief’s paradox*: both loss-related avoidance and loss-related approach behaviors appear implicated in the perpetuation of prolonged grief symptoms. We note that the clinical implications of these positions are different: if *only* loss-related avoidance perpetuates prolonged grief symptoms, then reducing such avoidance may be critical to treat PGD; if *only* loss-related approach perpetuates prolonged grief symptoms, then the disruption of the approach behaviors should be central to PGD treatments. To solve this paradox, it is critical to define what is avoided, what is approached, and how these processes are interrelated within PGD.

We propose that there are three interrelated reasons that have thus far obscured understanding of the role of avoidance and approach behaviors in prolonged grief. First, the cognitive behavioral model of prolonged grief does not hold that bereaved people avoid reminders of the loss per se, but rather that people avoid reminders of the permanence of the separation with the deceased (i.e. the reality of the loss) and associated negative emotions (Boelen et al., 2006b). While avoided aspects of the loss are often idiosyncratic, they are proposed to be share a single characteristic: they represent the fact that a loved one is ‘dead and gone’.

Second, multiple processes that on the surface appear to heighten focus on (i.e. approach) the deceased, such as yearning, proximity-seeking, and rumination, have been proposed to paradoxically serve as/be intrinsically linked with avoidance of the reality of the loss (e.g. Boelen et al., 2006b; Eisma et al., 2020b; Field et al., 2003). For example, rumination about why the loss occurred may take up cognitive resources to enable suppression of more threatening loss-related thoughts and memories (Eisma et al., 2013). Indeed, grief rumination is positively associated with avoidance of the reality of the loss in surveys and behavioral tasks (Eisma et al., 2013, 2014; Eisma, Rinck, et al., 2015), and exposure treatment has been shown to reduce grief rumination and prolonged grief symptoms in one randomized controlled trial (Eisma, Boelen, et al., 2015).

Third, processes such as yearning, proximity seeking, and rumination, may be self-perpetuating not only because they serve as loss-reality avoidance and can thereby (temporarily) reduce negative emotions but also because they may be rewarding in other ways. Close attachment bonds serve as an important source of comfort, security, and happiness. When death breaks this bond, the desire for closeness with the deceased loved one usually gradually subsides over time, and different persons will be sought to serve the attachment-related functions of the deceased (for a review: LeRoy et al., 2019). However, this does not appear to be the case for the minority of bereaved persons with severe and persistent grief. Among this group, a strong desire to remain close to the deceased persists and reminders of the deceased continue to have a reinforcing quality (cf. Kakarala et al., 2020). For these people, recurrent thinking about how the loss could have been prevented (rumination), imagining what it would be like to be reunited with the deceased (yearning), and doing things to maintain a bond with the deceased (proximity seeking), may enhance positive feelings, such as feeling loyal to and connected with the deceased.

Based on the above, we formulate the *Approach-Avoidance Processing Hypothesis*. It holds that PGD is characterized by multiple, repetitive behaviors that maintain a focus on (approach) the deceased, yet that could paradoxically serve as loss-reality avoidance. These behaviors are self-perpetuating because they (in the short-term) reduce the experience of negative emotions associated with the loss-reality and increase (some) positive emotions, such as feeling loyal to the deceased. In the long-term, such behaviors hamper processing of the loss, for example by limiting the elaboration of memories of the loss and integration of these memories within existing autobiographical knowledge (Boelen et al., 2006b).

Within the present study, we aimed to provide the first empirical test of the *Approach-Avoidance Processing Hypothesis*. Latent class analyses (LCAs) were used to identify latent groups of bereaved people demonstrating specific patterns of loss-related ‘approach’ behaviors (i.e. yearning, proximity seeking, and rumination) and avoidance behaviors (i.e. loss-reality avoidance, experiential avoidance). We hypothesized that we would identify a class with high probabilities of reporting approach *and* avoidance behaviors. Additionally, we expected to identify classes with behavior profiles marked by lower probabilities of reporting approach and/or avoidance behaviors. We predicted that higher prolonged grief symptom levels and higher prevalence of probable PGD according to ICD-11 and DSM-5-TR would characterize the high approach/high avoidance class. We considered symptoms of both types of PGD as a type of sensitivity analysis, since there are some differences between the two current versions of PGD (e.g. in number of symptoms and symptom content), which may imply that their phenomenological characteristics differ (Eisma et al., 2022).

## Method

### Participants

The Ethical Committee Psychology of the University of Groningen approved this study (registration number: PSY-1819-S-0173). We recruited Dutch adults who experienced the death of a loved one to participate in a survey on coping with bereavement via Google Ads. The survey was programmed in Qualtrics and could be accessed via the website www.onderzoekrouw.nl. All participants were provided with information about study aims and procedures (e.g. data handling, anonymity, voluntariness of participation) and gave online informed consent before starting the survey. The average time for completing the survey was approximately 30 min.

In total, 987 people started the online survey between May 2019 and September 2020. Of these participants, 817 completed all measures of interest to the present study. From this sample, we selected 288 participants whose loss occurred more than 12 months ago (in line with the time criterion of PGD DSM-5-TR and ICD-11). Answering all questions was required, so there was no missing data. Table 1 shows all sample characteristics. The majority of participants were female (92%), highly educated (54% attended university/college), and middle-aged (*M *= 52.76, *SD *= 13.96). Most had lost either a partner (46%) or a parent (27%) due to natural causes (75%). The average time since loss was approximately 33 months.

**Table 1.** Demographic and loss-related characteristics of sample (*N* = 288).

Sociodemographic variables	
Gender, *n* (%)	
Female	264 (92)
Age, *M* (*SD*)	52.76 (13.96)
Educational level, *n* (%)	
Primary school	1 (0)
Secondary school	72 (25)
Vocational School	59 (21)
College/University	156 (54)
Loss-related variables	
Deceased was, *n* (%)	
Partner, lover and/or spouse	132 (46)
Parent	79 (27)
Sibling	28 (10)
Child	36 (13)
Other	13 (5)
Cause of death, *n* (%)	
Natural (incl. disease and euthanasia)	217 (75)
Accident	24 (8)
Murder	2 (1)
Suicide	45 (16)
Time since loss, *n* (%)	
1–2 years	114 (40)
2–3 year	64 (22)
3–4 year	37 (13)
4–5 year	37 (13)
>5 years	36 (13)
Expectedness, *n* (%)	
Expected	80 (28)
Unexpected	157 (55)
Other (i.e. both or neither)	51 (18)

### Measures

#### Loss-related approach behavior

##### Rumination

Grief rumination, defined as repetitive and recurrent thoughts about the loss and related negative emotions was assessed using the Utrecht Grief Rumination Scale (UGRS: Eisma et al., 2012, 2014). The 15-item UGRS describes thoughts regarding emotional reactions to the loss, the injustice, meaning and consequences of the death, counterfactual thinking about the death, and thoughts about how others respond to the loss. Participants indicated how frequently they engaged in certain types of thoughts on a 5-point Likert scale ranging from 1 (never) to 5 (very often). An example item is ‘How often in the past month did you analyze if you could have prevented the death?’. Previous studies reported good psychometric properties of the UGRS (Eisma et al., 2012, 2014). In the current study, the UGRS showed excellent reliability (α = .90)

##### Yearning

Yearning is a repetitive, cognitive-emotional process characterized by unsatisfied, intense, and future oriented appetitive desire towards a lost person (Davis et al., 2003; Robinaugh et al., 2016). The current study captured this behavior by using the Yearning in Situations of Loss Short-Form (YSL-SF), an abbreviated, improved version of the original YSL (O’Connor & Sussman, 2014). The YSL-SF is comprised of eight items that capture affective and cognitive aspects of yearning (Eisma et al., 2020b). For example, the item “The feeling of wanting the deceased back is so strong it is indescribable” reflects the affective component of yearning while, for instance, the item “I find myself wishing things could be the way they were when I was with the deceased person” reflects the cognitive component of yearning. Participants indicated how often they had experiences indicative of yearning on a scale ranging from 1 (never) to 5 (very often). In the current study, the YSL-SF demonstrated good reliability (*α* = .89).

##### Proximity seeking

Proximity seeking can be conceptualized as behaviors that serve to maintain the memory of the deceased or to honor the deceased (cf. Shear, et al., 2007). We used the Proximity Seeking Behavior Scale (PSBS) to assess participants’ proximity-seeking behavior (Eisma & Nguyen, 2022). The PSBS encompasses six items reflecting different proximity seeking behaviors (e.g. “I watched or listened to things that remind me of what life was like with the deceased”). Participants had to rate their frequency of acting as described in each item over the past year on a five-point scale ranging from 1 (never/rarely) to 5 (very often). In the current study, the PSBS items showed good internal consistency (*α* = .83).

#### Loss-related avoidance behaviors

##### Anxious avoidance

The anxious avoidance subscale of the Depressive and Anxious Avoidance in Prolonged Grief Questionnaire (DAAPGQ) was used to assess anxious avoidance, the avoidance of confrontation with the reality of the loss (Boelen & Van den Bout, 2010; Eisma et al., 2013). The DAAPGQ Anxious Avoidance subscale comprises four items (e.g. “I avoid situations and places that confront me with the fact that [-] is dead”). Furthermore, with consent of the first author of the original scale, the answer scale of the original DAAPGQ was adjusted from eight to four points ranging from 1 (not at all true for me) to 4 (completely true for me). The main reason for doing so was to make the scoring options easier to understand. The original scale did not use anchors for points between the lowest (1) highest points (8) of the answer options, which might obscure the meaning of the answer options between these points (2–7). We added anchors for all answer options. The DAAPGQ has shown adequate psychometric properties in prior studies (Boelen & Van den Bout, 2010, Eisma et al., 2013). In this study, the anxious avoidance subscale of the DAAPGQ was shown to be reliable (α = .81)

##### Experiential avoidance

The Acceptance and Action Questionnaire II (AAQ-II) was used to assess experiential avoidance, defined as the attempt to alter the form, frequency, or situational sensitivity of difficult private events (i.e. thoughts, feelings, and physiological sensations). The AAQ-II is a revised version of the original acceptance and action questionnaire (AAQ, Hayes et al. 2004; Bond et al., 2011). In this study, a Dutch version of the questionnaire was used (Jacobs et al., 2008). The AAQ-II consists of ten items on which participants have to indicate their frequency of experiencing described thoughts or behaviors on a 7-point scale ranging from 1 (never) to 7 (very often) (e.g. “It is OK if I remember something unpleasant” (reverse scored)). We recoded the answers so that higher scores would indicate more experiential avoidance. In the current study, the AAQ-II showed excellent internal consistency (α = .92).

##### Prolonged grief symptoms

The Traumatic Grief Inventory Self-Report Plus (TGI-SR+: Lenferink et al., 2022a) is a 22-item self-report measure. The TGI-SR+ is based on the 18-item TGI-SR which can be used to screen for symptoms of PGD per Prigerson et al. (2009) and persistent complex bereavement disorder per DSM-5 (Boelen & Smid, 2017). The TGI-SR+ includes four additional items to assess symptoms of PGD per ICD-11 and DSM-5-TR. Participants indicate the extent to which they experienced the symptoms included in the questionnaire during the preceding month on a five-point scale ranging from 1 (never) to 5 (always) (e.g. “I experienced difficulty to move on with my life (e.g. pursue friendships, activities)”). In the present investigation, the TGI-SR showed excellent reliability (α = .95). The sum score of all TGI-SR+ items was used as a measure of prolonged grief symptoms.

The TGI-SR+ was also used to determine a probable diagnosis of PGD (per DSM-5 TR, ICD-11: see Lenferink et al., 2022a for scoring rules). Responses of 4 (frequently) and 5 (always) were rated as symptom present. Participants were considered to have probable PGD per DSM-5-TR when one criterion B item (item 1 or 3), at least three criterion C items (item 2, 6, 8, 9, 10, 11, 18, 19, and 21), and criterion D (item 13) were present. Participants were considered to have probable PGD per ICD-11 when at least one criterion B item (items 1 or 3), at least five criterion C items (items 2, 5, 8, 9, 10, 16, 19, 20, 21, and 22), and criterion E (item 13) were present.

### Statistical analyses

Zero-order Pearson correlations between the total scores of the questionnaires (UGRS, YSL-SF, PSBS, DAAPGQ, AAQ-II, TGI-SR+) used in this study were inspected in order to explore the relationships between the key constructs under investigation.

LCA was used to investigate the potential existence of subgroups among bereaved people that evidenced differential patterns of loss-related approach and avoidance behaviors. Aforementioned measures of loss-related approach and avoidance (UGRS, YSL-SF, PSBS, DAAPGQ, AAQ-II) were recoded into indicators. We chose to limit the number of indicators due to our relatively small sample size (Nylund-Gibson & Choi, 2018) by dichotomizing complete questionnaires assessing specific approach and avoidance behaviors. To derive a cut-off point for each questionnaire, the number of items of a questionnaire was multiplied by midpoint value of the response scales. This aligns with previous studies using symptom based LCAs that dichotomized participant responses. For instance, on a 5-point scale, items rated as 3 (sometimes), 4 (frequently), and 5 (always) would be considered to imply that a symptom is present (Nickerson et al., 2014). This approach seemed appropriate as each questionnaire captures a single construct, namely a single type of loss-related approach or avoidance behavior. Accordingly, total scores ≥ 45 on the UGRS were coded as “rumination present” whereas scores < 45 were coded as “rumination not present”. YSL total scores ≥ 24 were coded as “yearning present” and scores < 24 were coded as “yearning not present”. PSBS total scores ≥ 18 were coded as “proximity-seeking present”, whilst scores < 18 were coded as “proximity-seeking not present”. DAAPGQ total scores ≥ 10 were coded as “anxious-avoidance present” and scores < 10 were coded as “anxious-avoidance not present”. AAQ-II total scores ≥ 40 were coded as “experiential-avoidance present” whereas scores < 40 were coded as “experiential-avoidance not present”.

Latent Gold version 5.0 was used for the LCA (Vermunt, & Magidson, 2013). The optimal number of latent classes was evaluated by successively adding classes to the most parsimonious one class model, up to a six-class model. Model fit was judged based on the Bayesian Information Criterion (BIC), the Sample-Size Adjusted Bayesian Information Criterion (SA-BIC), and the Akaike’s Information Criterion (AIC). For these indices, a smaller value indicates better model fit (Nylund et al., 2007). Moreover, the entropy R^2^, a measure reflecting the confidence of model classification, was inspected. R^2^ values closer to 1 relate to higher confidence that people belong to a certain class (van de Schoot et al., 2017). Furthermore, bootstrap likelihood ratio tests (BLRt) were computed to investigate whether the addition of a certain class yielded significant improvements (*p *< .05) in terms of model fit compared to the model with one class less (Nylund et al., 2007). Beyond these fit parameters, class size and theoretical interpretation were considered. Solutions with relatively small classes were not preferred as they provide computational difficulties in examining correlates of class membership.

Finally, after determining the optimal class solution, potential differences in terms of prolonged grief symptomatology between the classes were investigated using the three-step method by Vermunt (2010). This approach utilizes maximum likelihood estimates in order to estimate associations between class membership and external variables, or in this case, the association between class membership and total scores on the TGI-SR+ and probable caseness of PGD per ICD-11 and DSM-5-TR.

## Results

### Preliminary analysis

Zero-order correlations between the total scores of the questionnaires used in this study are presented in Table 2. Significant moderate to strong positive bivariate correlations were found between most variables of interest. A notable deviation from this were the non-significant correlations of anxious avoidance and experiential avoidance with proximity seeking.

**Table 2.** Zero-order correlations between approach and avoidance behaviors and prolonged grief symptoms (*N* = 288).

	2	3	4	5	6	*M* (*SD*)
1. Rumination	.66*	.38*	.37*	.60*	.77*	42.72 (12.59)
2. Yearning		.46*	.41*	.50*	.74*	26.50 (7.41)
3. Proximity-seeking			-.11	.10	.33*	17.63 (5.85)
4. Anxious avoidance				.51*	.47*	7.63 (2.97)
5. Experiential avoidance					.76*	36.68 (13.65)
6. Prolonged grief symptoms						64.66 (19.07)

Note: * *p* < .01 (2-tailed).

### Classes of loss-related approach and avoidance behaviors

#### Selection of the optimal class solution

One- to six-class models were estimated. Table 3 shows the corresponding fit indices. The three-class model yielded the lowest BIC and highest entropy R^2^ value. Additionally, the significant BLRt indicated that the three-class model is preferred over the four-class model. The smallest class size of the three-class model (*n* = 79) was adequate. The non-significant BLRt *p*-value of the four-class model indicated that this model did not show better fit than the three-class model. All models yielded relatively low entropy values (*R*^2^ < .80), indicating limited class distinctiveness. Based on these findings, the three-class model was selected. To investigate possible reasons for the low entropy values, the discriminative ability of the individual indicators was tested for the three-class model. All indicators, except proximity seeking (*p* = .11), contributed significantly to the class distinction (all *p’*s < .001).

**Table 3.** Goodness of fit statistics for 1–6 class models of approach and avoidance behavior (*N* = 288).

Model	LL	SA-BIC	BIC	AIC	BLRt *p*-value	Entropy *R*^2^	Smallest class size
One-class	−946.80	1906.05	1921.91	1903.59	–	1	288
Two-class	−862.44	1752.29	1787.17	1746.88	<.001	.69	138
Three-class	−844.33	1731.02	1784.93	1722.66	.01	.71	79
Four-class	−834.06	1725.43	1798.37	1714.12	.60	.71	42
Five-class	−832.93	1738.12	1830.09	1738.12	.30	.64	39
Six-class	−831.35	1749.91	1860.90	1732.70	.42	.64	33

Note: LL, log-likelihood; SA-BIC, sample-size adjusted Bayesian information criterion; BIC, Bayesian information criterion; AIC, Akaike information criterion; BLRt, bootstrap likelihood ratio test.

#### Three-class solution

The conditional probability estimates of the different loss-related approach and avoidance behaviors across the different classes are presented in Table 4. Previous studies stipulated that probabilities below .15 can be considered low, rates of .60 or more as high, and those between these values as moderate (Burstein et al., 2012). Overall, the classes within the three-class solution can be roughly described as a low approach/low avoidance class (*n *= 98, 34%), a high approach/low avoidance class (*n *= 79, 27%), and a high approach/high avoidance class (*n *= 111, 39%). The low approach/low avoidance class presented a uniform pattern of results with conditional probabilities ranging from low to moderate across the indicators. The high approach/low avoidance class was characterized by high probabilities of yearning and proximity seeking and low conditional probabilities of experiential avoidance and anxious avoidance. The moderate conditional probability of grief rumination constitutes a minor deviation from this overall pattern. Finally, the high approach/high avoidance class was characterized by high probabilities across most approach and avoidance behaviors. A moderate probability of endorsing anxious avoidance was found within this group.

**Table 4.** Probability estimates of loss-related approach/avoidance across classes (*N* = 288).

	High Approach/high Avoidance (*n *= 111, 39%)		High Approach/low Avoidance (*n *= 79; 27%)		Low Approach/low Avoidance (*n *= 98; 34%)	
	Prob.	*SE*	Prob.	*SE*	Prob.	*SE*
Grief Rumination	.**81**	.05	.32	.07	.05	.04
Yearning	.**94**	.03	.**72**	.07	.27	.09
Proximity Seeking	.**60**	.05	.**83**	.15	.03	.06
Anxious Avoidance	.56	.06	.07	.05	.11	.04
Experiential Avoidance	.**85**	.06	.05	.07	.24	.06

Note: Values in bold indicate a high probability.

#### Association between class membership and prolonged grief symptoms

Table 5 shows analyses of covariates of class membership. Significant differences in prolonged grief symptoms were found between all classes using the three-step approach. Individuals in the high approach/high avoidance class on average showed the highest symptom levels (*M *= 81.87), followed by the high approach/low avoidance class (*M *= 59.48), and the low approach/low avoidance class (*M *= 46.69). Overall, 92 participants (32%) met the criteria for a probable diagnosis of PGD per DSM-5-TR. Of these participants, 82 (89%) belonged to the high approach/high avoidance class, eight (9%) to the high approach/low avoidance class, and two (2%) to the low approach/low avoidance class. Similarly, 81 participants (28%) with a probable diagnosis of PGD per ICD-11 were identified. Of these participants, 79 (98%) belonged to the high approach/high avoidance class, two (2%) to the high approach/low avoidance class, and zero (0%) to the low approach/low avoidance class.

**Table 5.** Univariate differences in prolonged grief symptoms and probable ICD-11 and DSM-5-TR PGD between classes (*N* = 288).

	High approach/high avoidance vs. High approach/low avoidance			High approach/high avoidance vs. Low approach/low avoidance			High approach/low avoidance vs. Low approach/low avoidance		
	B	SE	95% CI	B	SE	95% CI	B	SE	95% CI
Prolonged grief symptoms	−0.21	0.04	−0.14, −0.28	−0.29	0.04	−0.37, −0.21	−0.08	0.02	−0.12, −0.04
Probable PGD DSM-5-TR	−3.21	0.66	−4.50, −1.92	−4.25	0.99	−6.19, −2.30	1.04	1.20	−1.31, 3.39
Probable PGD ICD-11^a^	–	–	–	–	–	–	–	–	–

Note: The first-mentioned class is the reference category ^a^ Probable PGD per ICD-11 could not be compared with the 3-step approach because zero people had probable PGD in the Low approach/low avoidance class.

## Discussion

This study provided the first empirical test of the *Approach Avoidance Processing Hypothesis*, which holds that PGD is characterized by multiple, self-perpetuating, repetitive behaviors that maintain a focus on (approach) the deceased, yet paradoxically serve as loss-reality avoidance. The first aim of this investigation was to test whether a latent class of bereaved people could be identified with high odds of showing repetitive behaviors which appear focused on approaching the deceased (i.e. yearning, proximity seeking, rumination) co-occurring with avoidance of loss-related cues and related internal experiences (i.e. anxious avoidance, experiential avoidance). A second aim was to assess whether this subgroup, compared to groups with lower odds of engaging in approach and/or avoidance behaviors, would demonstrate higher prolonged grief symptom severity and a higher proportions of people with probable PGD per DSM-5-TR and ICD-11.

LCAs showed that a three-class solution provided the best fit for approach and avoidance behavior in a bereaved community sample. Thirty-four percent predominantly showed low probabilities of approach and avoidance behavior (i.e. low approach/low avoidance class), 27 percent showed predominantly high probabilities of approach behavior and low probabilities of avoidance behavior (i.e. high approach, low avoidance class), and 39 percent showed predominantly high probabilities of approach and avoidance behaviors (i.e. high approach, high avoidance class). The latter class consistently demonstrated the most severe grief reactions compared to other classes, indicated by higher prolonged grief symptom levels and higher odds to experience probable PGD per DSM-5-TR and ICD-11. The high approach/low avoidance class, in turn, demonstrated more severe prolonged grief symptoms than the low approach/low avoidance class, but did not significantly differ from this class on the odds of probable PGD.

Turning to the class solution first, three classes provided the most optimal solution to the latent class analysis considering all fit indices, class sizes, and interpretability of results. Additionally, most indicators significantly contributed to the distinction of the classes, indicating they added uniquely to the final class solution. However, entropy of the class solution was relatively low, implying limited distinctiveness of classes (van de Schoot et al., 2017). We attribute this finding to the fact that the high approach/low avoidance class showed comparable probabilities of yearning and proximity seeking to the high approach-avoidance class, as well as comparable probabilities of anxious and experiential avoidance to the low approach/low avoidance class. In other words, overlap on the conditional probability of experiencing approach and avoidance behaviors between the high approach/low avoidance class and other classes resulted in modest class distinctiveness.

Results appear generally consistent with the *Approach-Avoidance Processing Hypothesis of PGD*. For nearly forty percent of our sample, recurrent processes focused on retaining a connection with the deceased, such as yearning, proximity seeking, and grief rumination, were likely to co-occur with avoidance of loss-related cues and internal experiences. The fact that this pattern of approach and avoidance behaviors was strongly indicative of severe grief reactions supports the idea that simultaneous occurrence of approach and avoidance characterize PGD. It should be noted that the class solely demonstrating high odds of approach behaviors (particularly yearning and proximity seeking) also showed elevated prolonged grief symptoms compared to the class showing low odds of approach and avoidance behaviors. However, there were few people in this class with probable PGD.

Taken together, findings suggests that most people with PGD, yearn to be close to the deceased, engage in behaviors to retain a bond with the deceased and ruminate about events leading up to the loss (e.g. Currier et al., 2014; Doering et al., 2018; Eisma et al., 2020b). This may be reinforcing both by increasing feelings of loyalty and connectedness as well as by acting as paradoxical avoidance strategies, by reducing accessibility of thoughts and memories related to reality of the loss and associated negative emotions (cf. Boelen et al., 2006b; Eisma et al., 2020b; Field et al., 2003). Eventually, such behavior hampers processing of the loss, for example by limiting the elaboration of memories of the loss and integration of these memories within existing autobiographical knowledge (Boelen et al., 2006b). As noted, some people with probable PGD have high odds of yearning for the deceased and engaging in behaviors to maintain a connection with the deceased yet show less avoidance behavior. For these people, the inability to relinquish an attachment bond and direct attachment-related functions toward other people in their social environment may lie at the heart of their grief complications (LeRoy et al., 2019).

Results on the co-occurrence of approach and avoidance behavior in people with severe grief align with prior research showing that yearning and rumination are positively related to avoidance behaviors and prolonged grief symptoms in survey research (e.g. Hardt et al., 2022; Morina, 2011; Eisma et al., 2013, Eisma et al., 2020b). Similarly, it corresponds with laboratory studies showing links between rumination and loss-avoidance using behavioral tasks, such as eye-tracking tasks (Eisma et al., 2014, 2015). While this may imply that approach behaviors could act as avoidance in some circumstances (cf. Stroebe et al., 2007), there are also instances where approach and avoidance behaviors do not co-occur. One interesting finding in this regard is that proximity seeking to the deceased by engaging in continuing bonds behavior did not show a significant zero-order correlation with anxious avoidance and experiential avoidance. Possibly, such behavior occurs relatively independently from avoidance tendencies. This particular finding appears difficult to reconcile with the Approach-Avoidance Processing Hypothesis and suggestions that maintaining a strong bond with the deceased could be a way to avoid the permanence of separation from the deceased (e.g. Boelen et al., 2006b; Field et al., 2003). Another interesting observation was that not a single class showed high probabilities of anxious avoidance (although the high approach/high avoidance class did approach a high probability). One potential explanation may be that people showing most severe grief reactions experience anxious avoidance most often. While prolonged grief symptom levels were highest in the high approach/high avoidance class, some people in this class may not meet criteria for PGD.

Clinically, results appear to imply that exposure therapy may indeed be suitable for most people with severe grief reactions. This aligns with findings that a significant minority experiences clinically relevant change in prolonged grief symptoms following exposure (Boelen et al., 2007; Eisma, Boelen, et al., 2015). From a clinical perspective, it is also useful to note that people exclusively showing avoidance behaviors were not identified in our LCA. Sometimes it may be difficult for clients to disentangle approach and avoidance processes. To reduce resistance to exposure, it may be useful to discuss with clients how approach and avoidance behaviors can co-occur (e.g. “you may be longing for the deceased day and night, but nevertheless there could be aspects of the loss that you would rather not think about”). Or, alternatively, one could explain how approach and avoidance behaviors may be intertwined (“You are going over the events leading to the loss over and over again, but this could also be a way to avoid thinking about the fact that your loved one is dead and gone”). Our results additionally suggest that exposure therapy is potentially less helpful for a minority of distressed bereaved persons who strongly engage in behaviors focused on maintaining a bond with the deceased, but not avoidance. For them, stimulating behaviors that may help them reengaging with life as well as their social relationships may be more helpful. Accordingly, setting new life goals, and increasing engagement with valued activities through behavioral activation form key elements of some treatments for prolonged grief (e.g. Eisma, Boelen, et al., 2015; Papa et al., 2013).

### Strengths, limitations and directions for future research

This study has offered novel insights into prolonged grief by applying latent class analyses to loss-related approach and avoidance behaviors of a large bereaved community sample. Yet, we should consider some limitations to qualify the interpretation of these findings. First, compared to the general bereaved population (cf. Kersting et al., 2011) there was an overrepresentation of higher educated females, as well as violently bereaved people, with the latter resulting in relatively high prolonged grief symptom levels (Djelantik et al., 2020). Results may vary depending on sample characteristics, so we recommend a replication of this study among samples with different sample characteristics. Second, since the aim of the present study is to assess the characteristics and underlying processes of PGD a degree of content overlap between the indicators within the latent class analyses and our covariates was inevitable. This overlap is most striking for yearning and prolonged grief symptoms. Other indicators are not directly captured by the TGI-SR+ and/or the items used to assess symptoms of PGD per ICD-11 and DSM-5-TR. Third, our measure of prolonged grief symptoms, the TGI-SR+, was applied to assess prolonged grief symptoms and probable PGD caseness per ICD-11 and DSM-5-TR (Lenferink et al., 2022a), but formal diagnoses could not be established. When clinical interviews become available to assess these new conditions, it appears worthwhile to compare the levels of approach and avoidance behaviors (and their interrelationships) in people with and without PGD. Fourth, the cross-sectional design of our study precluded conclusions about stability of these classes over time and about the temporal relationships between approach and avoidance behaviors and prolonged grief symptoms. Using longitudinal analyses such as latent transition analysis and latent growth curve modeling may offer a viable way to examine to what extent the behaviors under investigation are consistent over time and predictive of more severe grief trajectories (e.g. Bonanno & Malgaroli, 2020; Lenferink et al., 2020, 2022b; Sveen et al., 2018). Fifth, within this study, we analysed self-reported avoidance. One can wonder to what extent people are aware of their own avoidance behavior. Using implicit or behavioral measures, such as approach avoidance tasks, that may capture people’s approach and avoidance tendencies more subtly could form an additional way to assess their role within PGD (e.g. Eisma, Boelen, et al., 2015; Maccallum et al., 2015). Lastly, our relatively small sample size implied that we had to simplify indicators by dividing total scale scores into scoring ‘high’ and ‘low’ on a particular variable and applying latent class analyses. Future research on larger samples may use specific items of approach and avoidance behaviors as indicators and could apply latent profile analyses to gain a more fine-grained understanding of this topic.

### Conclusion

Despite these limitations, the present study illustrated that behaviors that appear to serve to approach the deceased and behaviors focused on avoiding the reality of the loss and related emotions co-occur among a group of bereaved persons showing severe prolonged grief symptoms. Nevertheless, prolonged grief may also occur in a small group showing lower odds of avoidance behaviors and high odds of approach behaviors. Should future research, using longitudinal designs and laboratory-based assessments of approach and avoidance behaviors confirm these relationships, it appears worthwhile to explore the clinical implications of these findings. Tailoring PGD treatments based on the profile of loss-related approach and avoidance tendencies of the bereaved client may enhance their effectiveness.

## Data Availability

The data, syntax and output for this article are available via DataverseNL: https://doi.org/10.34894/XQUDWV.
